# ‘Do Not Become Pregnant’: Negotiating HIV-Affected Pregnancy and Abortion in Late-Twentieth-Century Scotland

**DOI:** 10.1353/hah.2024.a952499

**Published:** 2024

**Authors:** Hannah J. Elizabeth

## Abstract

This article tracks how the advice to terminate pregnancies became integral to the medical and social management of HIV among women in the UK, and considers how this shaped women’s experiences of HIV-affected pregnancy. Specifically, it traces how the advice that ‘at risk’ and HIV positive women should avoid pregnancy, and should terminate pregnancies, evolved in the last decades of the twentieth century. It asks how this advice shaped the experiences of pregnant people affected by HIV, and then finally traces how the advice to terminate pregnancies ebbed away. In doing so, the article explores how healthcare practitioners and the women under their care viewed HIV-affected pregnancy, and the possibilities of HIV-affected motherhood and natal families it conjured, excavating the myriad tensions which shaped the decision to terminate or continue a pregnancy affected by HIV.

## Introduction

In 1987 the British Medical Association published *AIDS and YOU*, a hefty booklet intended to comprehensively represent HIV and AIDS from a health perspective to the British public, especially those at risk of, or already living with, AIDS. The booklet warned women to ‘[a]void becoming pregnant if you have been at risk’, and told those already living with HIV certain things were ‘dangerous to others’ including having a baby.^1^ Later it stated more emphatically: ‘Do not become pregnant’. The booklet’s guidance joined a growing chorus of similar advice from the government, health authorities, healthcare workers, charities, activists and the media seeking to prevent HIV-positive women from becoming mothers to HIV-affected children. But, across the UK, women who had been ‘at risk’ and women living with HIV and AIDS did become pregnant. Those women who discovered their status during pregnancy were generally advised, where medically and legally possible (generally before 22 weeks of gestation), to terminate their pregnancies.^2^

This article tracks how the advice to terminate pregnancies became integral to the medical and social management of HIV among women in the UK and how this shaped women’s experiences of HIV-affected pregnancy and potential parenthood. To explore this history, the article examines the experiences of HIV-affected women living in Edinburgh particularly because, as the article demonstrates, the city loomed large in both medical and cultural understandings of HIV-affected motherhood. In doing so, the article explores how healthcare practitioners and the women under their care viewed HIV-affected pregnancy, available options, and the myriad tensions which shaped the decision to terminate or continue a pregnancy affected by HIV.

The article begins by sketching the medical landscape around HIV and pregnancy in Britain, before acknowledging the specificity of Scotland’s policy and practice around abortion, and outlining the early epidemiology of Edinburgh’s HIV epidemic. Tracking the emergence of a variety of healthcare interventions such as testing, specialist clinics and cohort studies, it demonstrates Edinburgh’s importance in shaping policy and practice around HIV-affected pregnancy in the UK and beyond.

The article then explores how Edinburgh’s importance within the medical landscape and peculiar epidemiology led to intense media scrutiny around its HIV-affected citizens, especially women and drug users. This publicity contributed to a number of interrelated narratives around HIV and pregnancy emerging in the UK, and ensured that debates around HIV and abortion became a national discussion. Included in this conversation were the women themselves, along with those who advocated for their right to choose and those in opposition to women’s reproductive rights – opposed even to the provision of the HIV test to pregnant people lest it encourage abortion. The final section explores women’s experiences, illuminating how medical advice and media chatter influenced the experience of HIV-affected pregnancy decision-making in practice.

## A Note on Sources

This article draws on a variety of sources to illustrate how HIV-affected women received and experienced advice to terminate pregnancies. This patchwork is reflective of the scattered and patchy archive which documents this contested intervention. Abortion remains taboo, and the archive generally documents the experiences of those who followed medical advice and avoided pregnancy, or continued rather than ended their pregnancies, and even this is a fragmented archive. Sources which document the experiences of women who terminated their pregnancies, while discussed below, are rarer still. Frequently, women’s decisions have rendered them anonymous within the archive, their names, occupations and the area of the UK they lived in scrubbed from the record to avoid the double stigma of HIV and abortion, or to protect their families. Sources include the press, the 2015 Penrose Enquiry (Scotland’s investigation into the impact of NHS provision of contaminated blood and blood products in the 1980s), and the English Infected Blood Enquiry, 2019. As the majority of women living with HIV did not speak to the press or give evidence to these enquiries we cannot assume that those who did left representative testimonies. However, their stories are echoed elsewhere in the archive and give colour to the clinical encounters we see sketched out in advice literature, pamphlets and medical guidelines. And so, while such traces offer imperfect and partial individual glimpses, they are also indicative of certain collective experiences, showing the emotional impact of advice, media scrutiny and societal stigma.

## The Medical Context for HIV-Related Termination Advice in Britain

While the decision ultimately lay in the hands of the pregnant person, medical advice to terminate potentially HIV-affected pregnancies was clear and widely disseminated through both educational texts and the newsprint media in Britain. Where women had already developed life-threatening AIDS defining illnesses such as neumocystis carinii pneumonia, this advice was strongest, but women with asymptomatic HIV were also advised to terminate their pregnancies. Some women who had unconfirmed HIV, but were suspected to have been exposed, were also offered this advice

In the early years of the AIDS crisis the recommendation to terminate was partly motivated by the belief that pregnancy would accelerate the speed at which HIV-positive women developed AIDS or would intensify the severity of AIDS defining illnesses. The advice was also motivated by concern for the foetus. In the early years of the crisis, mother to baby transmission was believed to occur in around 50 to 80 percent of births, with the prognosis for paediatric AIDS considered very poor.^3^ While later studies showed that in cases of asymptomatic HIV, pregnancy seemed to have no discernible bearing on the progression of the virus for the woman, and asymptomatic HIV had little effect on pregnancy outcomes, concern remained as to the effect of pregnancy upon those with symptomatic AIDS.^4^ Moreover, as knowledge about the transmission and progression of HIV in infants and children remained thin into the 1990s, many doctors believed that outcomes for the infant, if transmission occurred, were severe enough to warrant termination to avoid any risk and consequent future suffering.

For some, the prognosis of adults living with HIV and AIDS made childbearing and childrearing inadvisable regardless of vertical transmission rates, parenthood rendered an unfortunate casualty of the virus. Indeed, as the Glasgow doctor Mary Hepburn later recalled in an oral history interview, ‘the advice for us in obstetrics was if a woman was diagnosed as having HIV, we should advise her to have a termination and be sterilised immediately.’^5^ While this sterilisation advice does not seem to have appeared in education literature for HIV-affected women in Britain, it was discussed in the medical literature at the time, and put into practice in some countries with where HIV prevalence was recognised among women, such as the USA and South Africa.^6^

Despite the chorus of advice to avoid pregnancy and to terminate, many HIV-affected people in Britain chose to become pregnant or continue their pregnancies. Indeed, ‘at risk’ women’s decision making frequently proved more complex than doctors anticipated. As one 1990 article in the *British Medical Journal* summarised it:

Perinatal transmission of HIV occurs in 15-33% of pregnancies, so the criteria for termination are certainly met. Some doctors will find it strange, therefore, that infection with HIV seems to take second place to socioeconomic considerations in determining termination requests, with little difference in the request rate between women who are and are not infected in high risk groups.^7^

As many women proved undeterred by a positive HIV test, the newsprint media and medical press were replete with handwringing discussions about how to persuade these HIV-positive women to prevent and terminate their pregnancies. Equally voluble were responses from those determined to defend women’s right to choose, or those advocating against women seeking HIV tests at all, lest it increase abortion rates.^8^ The need for empathetic treatment, the weight of women’s bodily autonomy, and the tenability of the HIV-affected family was thus debated, with those women who were exposed to the virus through partner infidelity or medical accident often treated differently from those whose exposure was related to their own drug use. For HIV-positive women who carried their pregnancies to term, through choice or because their HIV status was confirmed too late to intervene, the advice to abort could not simply be ignored. The terminate diktat was too pervasive, and the fearful pessimism which lay behind it too powerful. Even in defiance, some HIV-positive women later spoke of the guilt and fear they carried far beyond the term of their pregnancies, worried they had done something wrong in becoming parents.

## Abortion in Scotland

Where the woman is less than 22 weeks pregnant and presents seeking termination of pregnancy …this is clearly justified under the 1967 Abortion Act because of the risk of fetal infection and the possibility that the woman’s health and life expectancy may be limited. … where she decides against termination despite ominous investigations, or if she is more than 22 weeks gestation, the pregnancy should continue.^9^

As the above 1988 quote discussing HIV and abortion makes clear, by the late 1980s maternal HIV positivity was considered medically and legally relevant to the provision of an abortion, but ultimately, supposedly, the decision rested with the person carrying the pregnancy. But as the mention of the 1967 Abortion Act and the ‘22 weeks gestation’ threshold belies, power over provision rested firmly in the hands of medical practitioners, and, in Edinburgh, was shaped by a specifically Scottish history of abortion.

While legal abortion existed within Scotland prior to 1967 under common law – with doctors able to act in ‘good faith’ and determine when the termination of a pregnancy was a necessity – the 1967 Abortion Act extended and formalised the criteria for a legal termination of pregnancy between the first and twenty-fourth week of gestation across England, Wales and Scotland.^10^ Implemented in 1968, the passing of the Abortion Act saw a steep rise in terminations within Scotland, as was the case in England and Wales.^11^ However, while abortion provision in England and Wales in the latter half of the twentieth century was increasingly typified by a mixed economy of providers (including private and charitable organisations), the majority of abortions within Scotland were provided within NHS facilities – some ninety-nine percent by 1974.^12^ The additional work which terminations represented engendered complaints from practitioners that while the passing of the act created more work, it did not generate the requisite additional resources required to meet this rise in demand for abortion services.^13^ While this NHS dominance might suggest a comprehensive service, eliminating the potential for profiteering that private clinics are at risk of, the true picture is one of patchy provision that frequently obstructed access.

Access to terminations was (and is) affected by the personal views of healthcare practitioners and their right to conscientious objection. Indeed, following patterns seen before the passing of the Act, communities of pro-choice and anti-abortion health workers persisted well into the 1970s around specific figures, institutions, towns and cities, creating areas where care could or could not be sought.^14^ Despite the passing of the Act, many pregnant people had to leave Scotland in search of an abortion in the 1970s, most often traveling to London, Liverpool and Birmingham, or to more liberal Scottish cities such as Edinburgh and Aberdeen.^15^ While it is unclear from the archive if those living with HIV in the 1980s had to travel more than others for later terminations, the labyrinthine and patchy system that affected access to abortions for all pregnant people forms an important context for any consideration of the history of this HIV-related healthcare. Not least because the status of HIV as an indication for termination was hotly debated, with sides drawn along religious, medical and humanist grounds.

## The Edinburgh Cohort – Clinical Encounters and Pregnancy Among Drug-Using Women in the ‘AIDS Capital’

Stately Edinburgh is now the Aids [*sic*] capital of Europe. The infection is no longer known as the ‘gay plague’ but is spreading at an alarming rate into the heterosexual community. …The epidemic is due mainly to widespread practice of heroin users sharing needles. …Female heroin addicts …have turned to prostitution to finance the habit so that the problem has already gone far beyond city boundaries.^16^

The retrospective testing of blood samples in 1985, collected from injecting drug users (IDUs) to monitor an earlier hepatitis outbreak, indicated that around 1,000 and 1,500 IDUs contracted HIV in Edinburgh between the summers of 1983 and 1984.^17^ By 1985, an estimated fifty percent of ‘known’ injecting drug users – meaning those engaged by social, medical, or criminal justice workers – were HIV positive and, as the above *Sunday Telegraph* quote makes clear, the media knew it.^18^ These drug users were generally young (a mean age of 25 at diagnosis), had been taking intravenous drugs since they were teenagers, and came largely from a background of multiple deprivation.^19^ While all the above statistics represent estimates, Edinburgh’s early recognition, and consequent varied and integrated response to the HIV crisis among IDUs, and the media’s intense scrutiny, allows us to examine this early history with a degree of confidence.

At Edinburgh City Hospital a number of innovative clinics were developed to meet the unique care needs of Edinburgh’s HIV-affected population, including the needs of HIV-positive drug users, former drugs users, and pregnant people. These clinics took an innovative holistic approach to caring for the HIV affected, meeting their needs as drug users and families. As discussed below, these clinics also allowed patients to be recruited into cohort studies examining HIV.

While studies scrutinising the effect of AIDS upon women had begun, research investigating HIV in pregnancy and children was still sparse in the mid-1980s, with knowledge pertaining to the effect of asymptomatic HIV sparser still. The Edinburgh City Hospital Cohorts offered a chance to study the effect of HIV, rather than AIDS, on women, pregnancy, and infant development, with results from the myriad studies this group consented to constructing a more optimistic picture of the virus’s effects pre and post-partum. The integrated nature of Scotland’s maternity and gynaecological services also meant epidemiological enquiries could include both ‘at risk’ women presenting at maternity services and those requesting terminations, creating a fuller picture of the emerging crisis and allowing comparisons to be made between the long-term health trajectories of those who continued or terminated pregnancies. These clinical encounters did more than gather new information that furthered epidemiological understanding; they shaped the experiences of women living with HIV in Edinburgh and beyond. Moreover, the importance of this accuracy of data’s effect on Edinburgh’s response to the AIDS crisis emerging in the Lothian region should not be under estimated; indeed there were benefits to the intense scrutiny that the HIV-positive drug users in Edinburgh experienced.

Since Edinburgh was a population identified early on in the UK’s HIV epidemic, the medical and epidemiological findings and the care regimes created to meet their needs had huge impacts beyond the Lothians, shaping national and international policy and practice. Indeed, care for HIV-positive drug users in the city extended to their inclusion in clinical trials, despite the myriad difficulties they presented in terms of adherence to protocols and potential drug interactions with continued street drug use. That said, while the city’s response had broad implications for policy, practice and research (which make it an important case study), the specificity of Edinburgh’s cohort of HIV-affected families must not be glossed. As many of the families supported in Edinburgh had a history of drug use, the advice and care they received, while shaped by clinical need, was marred by ongoing stigma and prejudice that can be seen reflected in both the medical and popular press accounts of their lives and prognoses.

With the scale of HIV among IDUs in the city recognised, a self-referral clinic supervised by Dr Ray Brettle (infectious disease consultant) was established at Edinburgh’s regional infectious diseases unit in Edinburgh City Hospital, providing both pre- and post-test counselling and antibody testing. The clinic began work on 16 October 1985, which coincided with the National Blood Transfusion Service’s commencement of the testing of all blood samples.^20^ Users could self-refer to the clinic, or be referred through contact with drug self-help groups, social workers, GPs or other hospitals.^21^ The clinic practised a policy of strict confidentiality, but encouraged patients found to be HIV positive to inform partners and GPs of their status. This policy was later adjusted to allow the disclosure of the test results to patients’ GPs with written consent, but contact tracing was never attempted; instead patients were encouraged to bring sexual partners and those they had shared needles with to the clinic.^22^

Brettle and his colleagues advocated a strategy of ‘risk or harm reduction’, arguing that in the face of HIV more ‘realistic’ goals should be sought than drug abstinence or sexual celibacy.^23^ Consequently, pre- and post-test counselling included the discussion of avoiding pregnancy and ‘risk reduction’ strategies and, in the case of a client being pregnant, the possibility of termination. Where clients were confirmed to be HIV positive, they were counselled with the aim of more specific HIV-related harm reduction, ‘with the initial emphasis on reducing the progression of the disease in the patient and spread of the virus in the community’; this included ‘advice on safe sex and safe drug use’ and, for women, ‘the risk of pregnancy’.^24^ Arguing it was necessary to do more than offer advice such as ‘avoid pregnancy’ or ‘use barrier contraceptives’, the clinic acted as ‘an outpost’ of the Edinburgh family planning service, ‘to offer on site advice on contraception and supplies [and] urgent referrals for termination of pregnancy’ when required.^25^ But providing such services, while important to ensure holistic care and access, was no guarantee of their use. Brettle, reflecting upon his work in the early days of the AIDS crisis in Edinburgh, later explained:

We were trying to discourage everyone from having kids, …obviously we were going, you know, ‘don’t have kids, you know, they’re bound to be HIV-infected. You mustn’t have kids.’ And they said, ‘RUBBISH, that person and that person and that person, they were all positive, they’ve had kids, their kids are fine. They don’t have the virus.’ And so, they were well ahead of us in terms of information sometimes, …the obstetrician we worked with, he said ‘it’s amazing how high the barrier has to be before women will agree to an abortion.’^26^

While Brettle spoke with an admiration for the resilience to pressure shown by his former patients, women’s resistance to termination advice, based on community knowledge rather than the medical literature, was discussed with bewildered frustration in the contemporary research literature at the time. While many doctors felt perturbed by this behaviour, the research which emerged from scrutinising Edinburgh’s HIV-affected communities would ultimately reinforce this more optimistic view of vertical transmission rates.

By 1991 over 400 of Edinburgh’s HIV-positive drug users attended the City Hospital clinic for HIV counselling, testing and general healthcare, allowing interested healthcare practitioners to study the population with a degree of ease not normally associated with this ‘hard to reach’ group. It also created the opportunity for direct health communication, with leaflets explaining HIV and AIDS specifically to drug users produced for the first time.

This point of scrutiny, education and healthcare was joined in 1985 by the enrolment of the majority of Edinburgh’s known HIV-positive mothers and their babies into the European Collaborative Study (ECS) via Edinburgh’s City Hospital’s dedicated Paediatric AIDS clinic, allowing the entire natal trajectory of HIV-affected women to be surveilled.^27^ Data emerging from this Edinburgh study, alongside other sections of the ECS, would demonstrate that rates of vertical transmission were far lower than previously thought, but the termination advice for HIV-affected women ebbed away slowly.

## Termination Counselling

Advice to terminate was delivered through a variety of routes, and guidance quickly emerged to help healthcare practitioners and social workers guide women towards a decision. Alongside medical institutions like the BMA, a number of hospitals, health authorities, charities and activist groups produced literature to guide both doctors and patients through the latest information about HIV, pregnancy and prognoses for HIV-positive infants. As was the case with other elements of HIV/AIDS care, information was shared between interested groups through emerging networks and organisations, and their conferences and publications. In the early 1990s this information was then consolidated when a flurry of counselling manuals emerged, drawn from the experience of those tasked with advising HIV-affected people on issues of fertility.^28^ While certain elements were similar, advice was generally tailored to meet the needs of specific HIV-affected groups, with families affected by haemophilia receiving different experiences of counselling to families affected by drugs for example. Activist groups from within HIV-affected communities also produced advice, with haemophiliac groups, women’s groups and queer groups working to inform patients and healthcare providers about specific needs and challenges.

Counselling literature generally offered an explanation of the latest scientific and medical advice, explanations as to why fertility was an issue affecting HIV-affected communities, and entreaties to those working in the field to practice non-judgmental care. While specific advice was given regarding the needs of different vulnerable groups (such as haemophiliacs and IDUs), some advice could be generalised to all HIV-positive or at-risk women. Emphasis was placed on leaving the decision to terminate or continue the pregnancy with the ‘mother or parents’, as one would expect, but texts also expanded on why drug-using women often presented for medical care late on in their pregnancies, and why HIV-positive people might wish to continue or seek a pregnancy. As one 1991 manual cautioned, ‘knowledge of HIV [positivity] is not an effective contraception’, and ‘counsellors should never assume that life with HIV is worse than no life.’^29^

Indeed counsellors were advised to expect some women’s desire to have a baby to increase when they acknowledged that their lives might be shortened by HIV. As the 1992 manual *Counselling Drug Users about HIV & AIDS* explained of intravenous drug users:

For drug-using women with low self esteem, having a child can seem like the one worthwhile thing she can do. …As a result of being HIV-positive, many IDUs have a sense of urgency added to their desire for a child. Having children (provided you are able to take care of them…) is something that is respected in our society.^30^

Other counselling advice explained these social factors in greater depth. As one Dundee-based drug services social worker explained:

the infected mother is not being perverse in wanting the child …some mothers do want a child, not for the child’s sake, but to uphold a strained ‘marital’ relationship, to escape from the mother’s family, or to please the family; the mother has to be allowed to make her decision. If the mother decides on termination then she will require bereavement counselling.^31^

Where women did decide to seek a termination, counsellors were tasked with explaining the rules by which terminations were governed, with time limits and mode of termination presented as important factors in the decision to terminate or continue a pregnancy.

Despite all the best efforts of counsellors to create a calm space where women could make their own decision, time created an unavoidable pressure towards one resolution or another. Alongside the UK-wide 24-week limit on terminations, there was also a general consensus that procedures after 16 weeks should be avoided, if possible, as they were felt to carry greater psychological and physical risk, especially where early labour had to be induced. This meant some women, found to be ‘at risk’ late in their pregnancies, had to decide whether to progress their pregnancies before their HIV status was known, as waiting on test results might place them beyond the termination window. These time pressures are likely to have been more acute for many drug-using women. For a variety of reasons, drug-using women often presented later at maternity services than non-drug-using women. This was both by mistake and by design. Amenorrhea is a common side-effect of heavy opioid use, leading some women to miss the early signs of pregnancy or to assume their drug use has made them less fertile.^32^ Additionally, the effects of drug use on lifestyle may reduce the ability to make and keep appointments, including those related to pregnancy. Studies also indicate that stigma associated with drug use during pregnancy, fears around being judged, and interventions by the statutory services such as the seizure of infants or encouragement towards drug abstinence, created obstacles to accessing antenatal services.^33^

Despite barriers to care, higher than average rates of abortion were typical among drug users, with HIV-negative ‘at risk’ women presenting for induced abortions at similar rates to their HIV-positive counterparts. Many HIV-positive women had also had abortions prior to their HIV diagnosis. Indeed studies indicated that HIV-positive women were no more likely to terminate their pregnancies than HIV-negative women deemed to be ‘at risk’ of HIV, and social factors, rather than biomedical concerns, were more likely to determine the decision to progress or terminate a pregnancy.^34^ The social vulnerabilities and the stigma associated with drug use during pregnancy and motherhood would seem to have presented a greater inducement to abortion then than an HIV diagnosis. However, research does suggest that the risks of HIV intensified the scrutiny and management of drug-using women, increasing access to harm reduction resources in some quarters, but also intensifying medical, social and media admonishments if they failed to manage their fertility or made the decision to seek parenthood.

If an ‘at risk’ or HIV-positive woman took the decision to terminate her pregnancy, counsellors were advised to be prepared to provide ongoing counselling:

mothers will require intensive and prolonged counselling. Many female addicts are young and have no children so with the possible termination of a pregnancy comes the realisation that they may never have children, that agreeing to termination now might mean all future pregnancies will also have to be terminated. For women from deprived families, then they often view a child as the one thing they can call their own selves, and for such women denial of child-bearing can lead to further emotional problems as they see their sexual identity being irrevocably damaged.^35^

While counselling was supposed to be part of the process of testing, diagnosis and termination or birth from as early as 1985, testimonies from patients, doctors and nurses indicate that its provision was patchy across the UK.

Moreover, there is evidence that women experienced pressure to remain childless or terminate pregnancies even after vertical transmission risk data showed improved prognoses and means of mitigating risks improved. In part this may have been the result of the media’s failure to report transmission rates or the increasing variety of successful mitigations accurately, leading to confusion and persistent pessimism.^36^ As late as 2010, stories appeared in the Scottish press which drew a link between an HIV diagnosis and an inevitable decision to terminate a pregnancy, with no information offered regarding likely transmission rates or prognoses for adults or infants.^37^ Luck appears to have played a significant role in how pregnant people considered to be at risk of HIV were dealt with, with patchy training resulting in variable services and outdated advice. Women in Edinburgh, where expertise had begun to concentrate, perhaps stood a better chance of encountering up-to-date advice and services than those living in more rural locales, but even so, experiences varied.

## Media Representations of Abortion as a Consequence of HIV

Pressure to terminate occurred beyond the clinical or counselling encounter. Media narratives about HIV and abortion hinged on ideas of HIV as a terminal future for fertility, mother and baby, and, where drug users were concerned, a general unsuitability for parenthood. Indeed, certain narratives about HIV-affected womanhood and motherhood appeared in the popular press so frequently that they shaped the experiences and expectations of other HIV-affected women. There were, however, variations in the degree of sympathy and blame meted out in reporting about the issue. Where women were represented as innocent victims, rather than culpable deviants, the choice to continue or terminate a pregnancy was often treated with a deal more sympathy by the media. And while Edinburgh was frequently dubbed Britain’s ‘AIDS Capital’ and drew much of the press’s focus, these stories were not bounded by the geography of the Lothians. For example, the 1986 *Daily Mail* article ‘Abortion for girl in AIDS tragedy’ explains that:

A YOUNG wife whose husband gave her and her unborn baby AIDS is to have an abortion tomorrow. The heartbroken woman, who fears that she may not have much longer to live herself, came to her decision after days of agonising when her husband broke the shattering news.^38^

The article went on to explain that the woman, Julie, a Roman Catholic, ‘had tried for six years to have a baby’ but that upon hearing the news she was pregnant her husband ‘revealed he had been having homosexual affairs for two years and had contracted AIDS’, necessitating her termination. Though short, the article included many aspects of a typical account of HIV interacting with pregnancy: the presumption that transmission was inevitable between husband and wife, and vertically, and a strong implication that termination of pregnancy was the preferred (if tragic) choice. It is also worth noting the interaction between HIV testing, abortion and religion gestured to here. Many churches spoke out against the termination of HIV-affected pregnancies, regardless of confirmed viral status, and some even decried the use of HIV tests on pregnant women as they felt these led to an increase in abortions.^39^

Generally, media sympathy for individuals was predicated on the degree of agency HIV-positive women seemed to have over their HIV status and pregnancy, hinging on if some culpable ‘risky’ behaviour could be detected in their past or present and if a termination was beyond reach. One HIV-positive mother quoted by the *Times* told the paper of her shock at discovering she was seven months pregnant:

It would have been sheer stupidity to even take the chance of becoming pregnant. I didn’t want a baby, but it was too late to have an abortion. The rest of the pregnancy was sheer hell because I was so frightened.^40^

The article, titled forebodingly ‘A child doomed to be an orphan’, though offering largely sympathetic coverage, reinforced the idea of HIV as incompatible with parenthood, confirming to readers that termination was the inevitable preferred choice.

In the first decades of the AIDS crisis, if HIV-positive women chose to become pregnant, especially where their serostatus was seen as self-inflicted, the media was even more damning. Thus Julie, wife of a cheating husband, garnered more sympathetic copy from the *Daily Mail* than the unmarried Heather, a ‘YOUNG mother-to-be [who] admitted yesterday that she deliberately became pregnant despite knowing her boyfriend has AIDS’. The article went on to report that Dr Stuart Glover, a communicable diseases consultant, ‘blasted’ the couple as ‘ignorant’, explaining that he felt ‘Anyone doing this is really playing Russian Roulette’.^41^

Women’s magazines generally documented experiences of women living with HIV in greater depth than the tabloid press, giving space to women’s testimonies regarding testing and decisions around fertility rather than the torrid tales of clandestine affairs and tragic abortions which typified *Daily Mail, Daily Express*, and *Daily Record* outputs. However, they still presented HIV as a serious obstacle to parenthood.

For example, *Company* magazine’s four-page 1990 article titled ‘How you really feel about being HIV positive’, offered long-form answers to questions about living with HIV from seven interviewees, including three women, two HIV positive, the other married to a man living with HIV. The article began by asking, ‘Why did you decide to be tested and what happened afterwards?’. The first respondent, Claire, explained that an episode of shingles prompted her to seek a test. She then recalled how her positive HIV test result was broken to her, indicating the absence of adequate post-test counselling, a focus on preventing transmission rather than the health of the patient, and a pressure to avoid pregnancies:

I was shown to see a doctor. He said, ‘Just promise me you won’t get pregnant – you’ve got antibodies to the Aids virus. If you have a baby you’ll die and so will your baby.^42^

Later the interviewers asked the women interviewees, ‘How do you feel about not being able to have children?’, implying that HIV was an absolute inevitable obstacle to parenthood. Claire responded:

It’s a big question that all women diagnosed HIV positive have to deal with. I didn’t want children anyway, but when I was diagnosed I felt that decision had been taken away from me. Now we’re learning that babies stand a very good chance of being born HIV negative. I think even if I did want a child it would be a very difficult decision to take because there would be a chance it could be born positive.^43^

Jane, married to Peter who was living with HIV, explained, ‘We looked into whether it would be possible for Peter’s sperm to be ‘cleaned’ of the virus. It’s not, so we’ve decided not to take the risk of having a child.’^44^

## Terminating and Avoiding HIV-Affected Pregnancies

While counselling manuals, leaflets, medical journals, policy papers and, to a lesser extent, media coverage tell us what the advice was, the experience of receiving it and in some cases acting on it, is harder to reach. In their evidence for the English Infected Blood Enquiry (IBE) Frankie and her husband, Mr A.N., explained how they were persuaded to terminate two pregnancies in 1985 and 1989, describing something of their experiences and the impact of these decisions on their lives. The first pregnancy was terminated because the suspicion was that Mr A.N. had HIV, and Frankie’s GP strongly advised her husband’s doctor to counsel against the continuation of the pregnancy. Writing without the couple’s knowledge, he explained to Mr A.N.’s GP:

I would consider that, in view of the current nationally agreed advice given to haemophiliac patients, together with the likelihood of [Mr A.N.] being positive for HTLV-III antibody (70% of our severe haemophiliacs are positive) that the possible risks of transmission of the HTLV-III virus from [Mr A.N.] to his wife and hence to a foetus is sufficiently likely to represent grounds for termination under the Abortion Act.^45^

While the couple never saw this letter between doctors until it was revealed as part of the IBE proceedings, they adhered to their doctor’s counsel and terminated the pregnancy. As Mr A.N. later recalled in oral evidence to the IBE:

We were just so frightened. We didn’t get any proper advice whatsoever. We were literally just like rabbits in headlights. It was more than advice about getting a termination; it was actually, ‘We are leading you down this path’, which we took.^46^

Both Frankie and her husband were tested for HIV before the termination, and while his test subsequently came back positive, Frankie’s result was negative. In 1985, data emerging from America regarding the prognoses of neonates exposed to HIV swayed many to err on the side of caution, advising terminations as Frankie and A.N.’s doctors did. But by 1989 when Frankie fell pregnant again, more optimistic data was emerging regarding transmission rates and HIV-positive infant prognoses. Early American data based on mothers and infants living with AIDS was being supplemented by studies on mothers and infants with asymptomatic HIV, such as data from Edinburgh’s cohort studies,^47^ and the wider European Collaborative Study. This more optimistic Scottish data was making newspaper headlines, with the *Telegraph* announcing that 34 enrolled ‘Scottish Super Babies Are Beating Aids Virus’. The article quoting a cheerful Dr Mok: ‘It is almost embarrassing when I tell them [New York paediatricians] that only two of ours are ill, and even they do not have the full disease.’^48^ The article also quoted Roger Kent, Director of Social Work for the Lothian Region, who said, ‘You can’t help feeling hopeful, but you dare not say so’. This sentiment echoed a caution that was present in the medical literature even as Edinburgh’s results were discussed and the link between symptomatic HIV and higher rates of transmission was more firmly established.^49^

When in 1989 Frankie discovered she was pregnant again, and quite far along, frightened, she went to her GP for advice. In her written statement for the IBE she explained:

I was a frightened young woman who went to my GP for help and I was made to feel like I had no other option than to terminate my pregnancy. I can no longer even comprehend what was said to me to make me agree with what they wanted. … I was treated by the nurses like I was a murderer, like what I was doing was completely and utterly wrong.^50^

Notably after both terminations Frankie received no immediate counselling about HIV or the loss of her wanted pregnancies.

Experiences such as Frankie and Mr A.N.’s do not appear to be isolated. In a confidential statement to the Penrose Enquiry, one ‘patient’ noted how he and his wife decided to terminate a pregnancy because of his HIV status. The Penrose Report explained:

in denial after being diagnosed with HIV, …he continued to have unprotected sex with his wife. She became pregnant and was advised that the baby could have HIV and would not have a good chance of survival. She underwent a termination of the pregnancy. During this procedure his wife was treated as infectious and kept in a side room in the hospital. This experience was ‘devastating’ for his wife.^51^

It is unclear in this case if the advice to terminate was given alongside an HIV diagnosis. Certainly, women were advised to terminate pregnancies if their partner received a positive diagnosis, in part because in the early days of the epidemic the processing of HIV tests was a slow process and the provision of a termination was time sensitive. The isolation of this women, ‘treated as infectious and kept in a side room’, mirrors many of the negative birth experiences of HIV-affected women who chose to continue their pregnancies. Indeed, the alienating experience of early infection control procedures, which often involved isolating patients and barrier nursing, took an emotional toll on both those who continued and those who terminated their pregnancies.

## Conclusion

The advice to terminate HIV-affected pregnancies shaped the experiences of many women affected by the AIDS crisis in Britain in the 1980s and 1990s. As this article has demonstrated, women did not need to follow or even be directly advised to terminate a pregnancy to be affected by this advice, so pervasive was it within medical practice and popular perceptions of HIV-affected pregnancy. While the prognosis and advice around HIV-affected pregnancies improved over this period, as more was learnt about paediatric HIV, changes were uneven, and earlier fear and pessimism persisted.

These changes and experiences have been explored in this article in part through the important case study of Edinburgh, a city which looms large in the British history of HIV epidemiologically, but also because it was home to research and activism that shaped policy and practice nationally and internationally. The emphasis on the provision of abortion as a response to HIV infection among pregnant women in Edinburgh was intimately connected to the nature of the city’s HIV epidemic. While at first there were thought to be medical reasons why HIV-positive women should be advised to terminate their pregnancies, the social context of many of these women’s pregnancies must be considered of equal importance. Drug-using women were already a stigmatised and vulnerable group, their pregnancies viewed with caution, and their identity as patients and potential parents as problematic. HIV added another layer of stigma and vulnerability to their lives as patients and potential parents. Despite this, HIV-affected women in Edinburgh defied the termination advice, and through their parenthood and participation in Edinburgh’s important cohort studies, helped shape more optimistic approaches to HIV-affected families and family making.

## Footnotes

1 David Morgan, ed., *AIDS AND YOU: An Illustrated Guide*, 1st ed. (London: The British Medical Association, 1987), 14.

2 Helen Coyle, “A Tale of One City: A History of HIV/AIDS Policy-Making in Edinburgh, 1982–1994” (PhD thesis, University of Edinburgh, 2008), 118–20.

3 Jacqueline Mok and Helen Zealley Interviewed by Louise Williams, 3 July 2018, Acc18/017, Lothian Health Services Archive (hereafter LHSA), University of Edinburgh.

4 Frank D. Johnstone et al., “Does Infection with HIV Affect the Outcome of Pregnancy?”, *British Medical Journal* 296, no. 6620 (1988), 467; Frank D. Johnstone, “HIV and Pregnancy”, *BJOG: An International Journal of Obstetrics & Gynaecology* 103, no. 12 (1996): 1184–90; Frank D. Johnstone, Lorna Willox, and Ray P. Brettle, “Survival Time after AIDS in Pregnancy”, *BJOG: An International Journal of Obstetrics & Gynaecology* 99, no. 8 (1992): 633–36.

5 Mary Hepburn Interviewed by Debra Hearne, 22 May 2018, C1759/46, British Library Sound Archive.

6 V.T. Raziano et al., “Factors Associated with Sterilization among HIV-Positive US Women in an Urban Outpatient Clinic”, *AIDS Care* 29, no. 5 (2017): 612–17; Zaynab Essack and Ann Strode, “‘I Feel like Half a Woman All the Time’: The Impacts of Coerced and Forced Sterilisations on HIV-Positive Women in South Africa”, *Agenda* 26, no. 2 (2012): 24–34.

7 Stephen Norman, John Studd, and Margaret Johnson, “HIV Infection In Women: Needs Early Identification To Limit Complications”, *British Medical Journal* 301, no. 6763 (1990): 1231.

8 Claire Dover and Mike Atchinson, “Mothers-to-Be May Face Screening Checks”, *Daily Express*, 24 March 1987; Johnathan Petre, “Pregnancy Screening for Aids Decried by Cardinal Hume”, *The Daily Telegraph*, 3 April 1987.

9 F.D. Johnstone, “Termination of Pregnancy”, in *AIDS and Obstetrics and Gynaecology*, edited by Frank Sharp and Christopher Neville Hudson (London: Springer, 1988), 154.

10 Gayle Davis and Roger Davidson, “‘A Fifth Freedom’ or ‘Hideous Atheistic Expediency’? The Medical Community and Abortion Law Reform in Scotland, c.1960–1975”, *Medical History* 50, no. 1 (2006): 35; Gayle Davis, “The Medical Community and Abortion Law Reform: Scotland in National Context, c. 1960-80”, in *Lawyers’ Medicine: The Legislature*,*The Courts and Medical Practice, 1760-2000*, edited by Imogen Goold and Katherine Kelly (Oxford: Hart, 2009), 143–65; Gayle Davis and Roger Davidson, “‘Big White Chief’, ‘Pontius Pilate’, and the ‘Plumber’: The Impact of the 1967 Abortion Act on the Scottish Medical Community, c.1967–1980”, *Social History of Medicine* 18, no. 2 (2005): 283–306.

11 D. Munday, C. Francome, and W. Savage, “Twenty One Years of Legal Abortion,” *British Medical Journal* 298, no. 6682 (1989), 298.

12 Gayle Davis et al., “All Aboard the Abortion Express: A Historical Geography of the 1967 Abortion Act”, in *Abortion Across Borders: Transnational Travel and Access to Abortion Services*, edited by Christabelle Sethna and Gayle Davis (Baltimore: Johns Hopkins University Press, 2019), 122.

13 Davis and Davidson, “‘Big White Chief’, ‘Pontius Pilate’, and the ‘Plumber’”, 287–88.

14 Davis et al., “All Aboard the Abortion Express”, 120–21.

15 *Ibid*., 120–24.

16 Carole Dawson, “Babies of the ‘Aids Capital’”, *The Sunday Telegraph*, 13 April 1986.

17 J.R. Robertson et al., “Epidemic of AIDS Related Virus (HTLV-III/LAV) Infection among Intravenous Drug Abusers”, *British Medical Journal* 292, no. 6519 (1986): 527–29.

18 Judith Bury, Val Morrison, and Sheena McLachlan, eds, *Working with Women and AIDS: Medical, Social, and Counselling Issues* (London: Psychology Press, 1992), 10–12.

19 A. Chiswick et al., “The Edinburgh Cohort of HIV Positive Drug Users: Who Are They and Who Cares for Them?”, *AIDS Care* 4, no. 4 (1992): 421–24.

20 R.P. Brettle et al., “Human Immunodeficiency Virus and Drug Misuse: The Edinburgh Experience”, *British Medical Journal* 295, no. 6595 (1987), 421.

21 *Ibid*.

22 *Ibid*., 422.

23 *Ibid*.

24 *Ibid*.

25 *Ibid*.

26 *Workshop 2 Scotland in the Global: HIV through Injecting Drugs and Beyond*, Scotland in the Global (University of Edinbrugh, 2021), 2, https://www.youtube.com/watch?v=PNAbCwFIlL4.

27 For a more extensive exploration of this clinic, see Hannah J. Elizabeth, “Recovering Mothers’ Experiences of HIV/AIDS Health Activism in Edinburgh, 1983–2000.”, in Janet Weston and Hannah J. Elizabeth (eds) *Histories of HIV/AIDS in Western Europe* (Manchester: Manchester University Press, 2022), 164-191

28 Bury, Morrison, and McLachlan, *Working with Women and AIDS*; Lorraine Sherr, *HIV and AIDS in Mothers and Babies: A Guide to Counselling* (Oxford: Blackwell Scientific Publications, 1991); G. Mulleady, *Counselling Drug Users about HIV & AIDS* (Oxford and Boston: Blackwell Scientific Publications, 1992); Robert Bor et al., *Theory and Practice of HIV Counselling: A Systemic Approach* (London: Cassell, 1992).

29 Sherr, *HIV and AIDS in Mothers and Babies*, 91–92.

30 Mulleady, *Counselling Drug Users about HIV & AIDS*, 119.

31 Paul Lockley, “Social Work Counselling for A.I.D.S. Virus Positive Drug Addicts”, n.d., GD1/85/2/55, LHSA.

32 Marcia Jackson and Hilary Klee, “Practitioner Views of Pregnant Drug Users,” in *Drug Misuse and Motherhood*, edited by Marcia Jackson, Hilary Klee, and Susan Lewis (London: Routledge, 2002), 105–7.

33 Hilary Klee, “Antenatal Care: Expectations and Experiences”, in *Drug Misuse and Motherhood*, 63–77.

34 Judy Bury, “Pregnancy, Heterosexual Transmission and Contraception”, in *Working With Women and AIDS*, 47; F.D. Johnstone et al., “Women’s Knowledge of Their HIV Antibody State: Its Effect on Their Decision Whether to Continue the Pregnancy”, *British Medical Journal* 300, no. 6716 (1990): 23–24.

35 Lockley, “Social Work Counselling for A.I.D.S. Virus Positive Drug Addicts”.

36 “Fertile with Error,” *The Times*, 15 May 1996.

37 Gordon Mcilwraith, ‘Mum-to-be aborted twins after discovering lover had given her HIV’, *Daily Record, 20 January 2010, https://www.dailyrecord.co.uk/news/scottish-news/mum-to-be-aborted-twins-after-discovering-1048224 (Date Accessed 11/11/2024*

38 “Abortion for Girl in AIDS Tragedy”, *Daily Mail*, 8 December 1986.

39 Petre, “Pregnancy Screening for Aids”.

40 Jeremy Laurance, “A Child Doomed to Be an Orphan”, *The Times*, 1 Apri 1993.

41 Paul Smith, “AIDS Baby Gamble”, *Daily Mail*, 27 February 1987.

42 Martin Nicholas and Joanne Williams, “How You Really Feel about Being HIV Positive”, *Company*, June 1990.

43 *Ibid*., 87.

44 *Ibid*.

45 Letter quoted during Mr AN Infected Blood Enquiry oral evidence: “Regarding Mr AN’s Wife Frankie”, Infected Blood Enquiry, 1 August 1985; Mr AN, Frankie [ANON], and Amanda Beesley, Infected Blood Enquiry Transcript - London - 16 October 2019, 12–14, https://www.infectedbloodinquiry.org.uk/search?keywords=W1387.

46 Mr AN, Frankie [ANON], and Amanda Beesley, Infected Blood Enquiry Transcript - London - 16 October 2019, 35.

47 Jacqueline Mok et al., “Vertical Transmission of HIV: A Prospective Study”, *Archives of Disease in Childhood* 64, no. 8 (1989): 1140–45.

48 David Wastell, “Scottish Super-Babies Are Beating Aids Virus”, *The Sunday Telegraph*, 20 September 1987.

49 Jacqueline Mok, “Follow-Up of the Child Born to an HIV-Infected Woman”, in *HIV Infection in Children: A Guide to Practical Management*, edited by Jacqueline Mok and Marie-Louise Newell (Cambridge: Cambridge University Press, 1995), 155–76.

50 Infected Blood Enquiry Written Statement of Frankie WITN1388001, https://www.infectedbloodinquiry.org.uk/evidence/written-statement-frankie-anon

51 George William Penrose and APS Group Scotland, *The Penrose Inquiry: Final Report* (Edinburgh, 2015), http://www.nls.uk/scotgov/2015/9780857590220.pdf; “Penrose Inquiry”, accessed 30 March 2021, https://www.penroseinquiry.org.uk/index.html.

